# SnapEnsemFS: a snapshot ensembling-based deep feature selection model for colorectal cancer histological analysis

**DOI:** 10.1038/s41598-023-36921-8

**Published:** 2023-06-19

**Authors:** Soumitri Chattopadhyay, Pawan Kumar Singh, Muhammad Fazal Ijaz, SeongKi Kim, Ram Sarkar

**Affiliations:** 1 Department of Information Technology, Jadavpur University, Jadavpur University Second Campus, Plot No. 8, Salt Lake Bypass, LB Block, Sector III, Salt Lake City, Kolkata, 700106 West Bengal India; 2 Department of Mechanical Engineering, Faculty of Engineering and Information Technology, The University of Melbourne, Grattam Street, Parkville, VIC 3010 Australia; 3 National Centre of Excellence in Software, Sangmyung University, Seoul, 03016 Korea; 4 Department of Computer Science & Engineering, Jadavpur University, Kolkata, 700032 India

**Keywords:** Cancer, Health care, Engineering, Mathematics and computing

## Abstract

Colorectal cancer is the third most common type of cancer diagnosed annually, and the second leading cause of death due to cancer. Early diagnosis of this ailment is vital for preventing the tumours to spread and plan treatment to possibly eradicate the disease. However, population-wide screening is stunted by the requirement of medical professionals to analyse histological slides manually. Thus, an automated computer-aided detection (CAD) framework based on deep learning is proposed in this research that uses histological slide images for predictions. Ensemble learning is a popular strategy for fusing the salient properties of several models to make the final predictions. However, such frameworks are computationally costly since it requires the training of multiple base learners. Instead, in this study, we adopt a snapshot ensemble method, wherein, instead of the traditional method of fusing decision scores from the snapshots of a Convolutional Neural Network (CNN) model, we extract deep features from the penultimate layer of the CNN model. Since the deep features are extracted from the same CNN model but for different learning environments, there may be redundancy in the feature set. To alleviate this, the features are fed into Particle Swarm Optimization, a popular meta-heuristic, for dimensionality reduction of the feature space and better classification. Upon evaluation on a publicly available colorectal cancer histology dataset using a five-fold cross-validation scheme, the proposed method obtains a highest accuracy of 97.60% and F1-Score of 97.61%, outperforming existing state-of-the-art methods on the same dataset. Further, qualitative investigation of class activation maps provide visual explainability to medical practitioners, as well as justifies the use of the CAD framework in screening of colorectal histology. Our source codes are publicly accessible at: https://github.com/soumitri2001/SnapEnsemFS.

## Introduction

**Figure 1:**
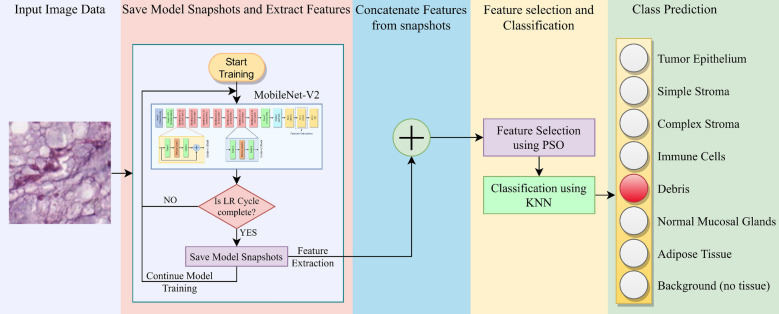
Overall framework for the detection of colorectal cancer from histology images. The Learning Rate (LR) cycle refers to the cycle intervals according to the cosine annealing learning rate scheduler used in this study. The model training continues until the maximum number of epochs is reached, while the features are extracted from the penultimate fully connected layer of the CNN model (refer to section “Transfer learning-based snapshot model training” for further explanation).

Colorectal cancer (CRC)^1^ is caused by the growth of polyps on the inner lining of the colon (or rectum) which is primarily due to mutation in certain genes that trigger an uncontrollable cell division, causing malignant cells to rapidly grow throughout the layers of the rectum as well as into the blood or lymph vessels. Being one of the leading causes of death in both developing and developed nations with a mortality rate of nearly $33.33\%$, the lifetime risk of developing CRC is about $4.3\%$ among men and $4.0\%$ among women^2^. However, early detection and diagnosis of this disease can increase the survival rate by almost $90\%$^3^, which stresses the requirement of a robust framework for the same. The conventional medical practices to detect CRC include stool-based tests and structural analysis of spectral imaging of colon and rectum linings^4^. However, stool-based tests only provide results for a limited time and therefore, needs to be done more often, while visual screening is prone to subjective variability as it is highly dependent on the observational capabilities of the pathologists^5^. Thus, an automation-assisted Computer-Aided Diagnosis (CAD) framework for histological analysis becomes a pressing need for robust detection of CRC while it is still in its early stages to prevent the spread of the disease to other vital organs.

Machine learning-based methods have largely been used in the past for the detection of CRC like the methods proposed by^6–8^. However, traditional machine learning methods require handcrafted features to be extracted from the image data. Such methods fail for complex pattern recognition tasks and it is also difficult to design a set of features that can accurately represent varied kinds of data.

On the contrary, deep learning methods^9–11^ alleviate the problem of traditional machine learning methods by performing end-to-end classification. Deep learning-based methods, especially Convolutional Neural Networks (CNNs) have become popular for medical image diagnosis tasks because of their ability to learn relevant features automatically from the input data. However, CNNs require a large amount of training data for achieving desirable performance which is often expensive to obtain, especially in the biomedical domain. Transfer learning is one of the solutions to this problem, where a model trained on a large dataset such as ImageNet^12^ is reused on the current problem containing a small dataset. In this study, we use the MobileNet-V2^13^ CNN model pre-trained on the ImageNet^12^ dataset that contains 14M images belonging to 1000 classes.

Ensemble learning^10,11,14^ is a fusion strategy that amalgamates the salient properties of multiple models and is popularly used in literature. However, ensembling two or more deep learning models can be a very computationally costly operation since each model needs to be trained separately for the fusion. This limits its use in many practical applications where there is a resource constraint. To this end, in this paper, we explore a different approach, where we perform an ensemble of snapshots of the same CNN model. That is, the CNN model is trained only once, and during its training, model snapshots are saved at different checkpoints. We extract features from the model snapshots (from the CNN model’s penultimate fully connected layer) and fuse them. However, since the features are extracted from the same CNN model (MobileNet-V2 in this case) at different training epochs, there must be some degree of redundancy in the overall feature vector. This is because though we take different snapshots, however, all the CNN models (here snapshots) will try to learn some common features from the data fed to the model. Hence, to eliminate the redundancy and to retain the important features only, we use a swarm-intelligence based meta-heuristic, called Particle Swarm Optimization (PSO) algorithm^15^, which in turn, selects the optimal subset of features from the feature space. This also reduces the storage requirements of the framework and makes the overall model computationally more efficient. We evaluate the performance of the proposed framework on the CRC detection problem using the publicly available dataset prepared by^16^, where our model is seen to outperform many state-of-the-art methods. Furthermore, we employ gradient-weighted class-activation mapping (Grad-CAMs), a weakly supervised segmentation algorithm^17^ that produces a heat map corresponding to the activations of the last convolutional layer, followed by its superimposition on the original histological image (please refer to section “Analysis of the proposed model” for a lucid explanation). This highlights the class-discriminative regions of interest found by our proposed model, which can of great help to medical practitioners in their decision-making process, making our framework a very useful CAD tool that can be easily leveraged in real-world prognosis.

The overall workflow of the proposed method is shown in Fig. 1. Specifically, our key contributions may be summarized as follows:

1. An ensemble learning-based deep FS framework is proposed for efficient and improved classification of CRC histological images.
2. Snapshot ensemble technique^18^ is adopted which allows using an ensemble of neural networks at the cost of training a single CNN model only.
3. PSO^15^ algorithm is used to perform FS on the fused feature set to choose the most relevant features from the feature space, thereby enhancing the performance of the framework as well as reducing storage requirements.
4. The proposed model is evaluated on a publicly available CRC histology dataset^16^ using a 5-fold cross-validation scheme and compared with several state-of-the-art methods, justifying its reliability and robustness for the CRC detection.
5. Last but not the least, the present work also investigates into Grad-CAMs obtained by the proposed model, which provide visual explainability and justification of the CAD pipeline that can aid medical practitioners in localising the region(s) of interest for medical prognosis.

The rest of the paper is organized as follows: Section “Related work” provides a comprehensive literature survey pertaining to the development of state-of-the-art in the domain of CRC detection; section “Proposed method” presents a detailed description of the proposed framework for the classification of histological images for CRC detection; section “Results and discussion” evaluate the proposed method on a publicly available dataset and provides a comparative study to establish the superiority of the method with respect to existing methods in the literature; and finally, section “Conclusion” concludes the findings from this research.

## Related Work

The development of a robust framework for the diagnosis of CRC is essential owing to its high mortality rate and widespread prevalence. One of its facets includes texture analysis of histological images^16,19–22^, which typically consist of an image pre-processing step followed by feature extraction, after which classification is performed using traditional machine learning algorithms. The handcrafted features may be extracted using texture filters such as Haralick^23^, Gabor^24^ and Local Binary Pattern (LBP)^25^, to name a few. Extracted LBP features from digitized colorectal tissue microarrays and trained a support vector machine (SVM) to classify between epithelium and stroma based on texture^19^. Studied multispectral imagery analysis of CRC histology biopsy samples and achieved an accuracy of $91.3\%$ by training an SVM classifier with LBP features^20^. Proposed a texture analysis framework for CRC detection by extracting features from multispectral optical microscopy images based on Laplacian-of-Gaussian (LoG) filter, discrete wavelets (DW) and grey level co-occurrence matrix (GLCM) based features^21^. Feature selection (FS)^26–29^ methods have also been leveraged in literature to select the most relevant features from the original feature set, extracted by some means, for improving upon the textural classification task. Performed spatial analysis of colon biopsy samples using Circular LBP features followed by a novel clustering-based FS method; the selected features were then used for training an SVM for classification, obtaining an accuracy of $90\%$^30^. The authors of^31^ performed FS using the Genetic algorithm on the fractal, curvelet coefficients and Haralick textural features from H &E stained histological images and reported an accuracy of $90.82\%$^31^. The authors of^32^ proposed a clustering-based modified Harmony Search Algorithm^33^ for FS on a CRC gene expression dataset, achieving an accuracy of $94.36\%$. However, these approaches may not be suitable enough for practical use as they were focused on the binary classification of tissue types, whereas histological images generally comprise multiple categories. The first study on multi-class texture separation of colorectal tissues was proposed by^16^, which presented a new dataset of 5000 histological images of human CRC including 8 tissue categories (described in Table 1)—the dataset we have used in this research. The authors performed classification by training several classifiers using multiple textural features, obtaining the highest accuracy of $87.4\%$ on the multi-class classification task.

**Table 1:** Classes of images in the CRC histology dataset^16^ used in this research.

Class	Category
0	Tumour epithelium
1	Simple stroma
2	Complex stroma
3	Immune cells
4	Debris
5	Normal mucosal glands
6	Adipose tissue
7	Background (no tissue)

Each class contains 625 images.

Deep learning methods, on the other hand, do not require handcrafted features to be extracted from the input images; rather, they exhibit self-learning behaviour in which the model learns the relevant informative features from the inputs by itself. This is particularly useful if the input data have very complex underlying patterns which are often difficult to capture using handcrafted feature engineering, as in the case of CRC tissue texture investigation. As such, CNN based models are preferred for image analysis as they can efficiently extract translationally invariant features from image data using blocks of convolution filters. The authors of^34^ proposed a bi-linear CNN-based model to extract and fuse features from stain decomposed histological images and obtained a multi-class classification accuracy of $92.6\%$ and sensitivity of $92.8\%$^34^. The authors of^35^ investigated the importance of stain normalization pertaining to tissue classification using a CNN model but achieved a classification accuracy of only $79.66\%$. Raczkowski et al.^36^ introduced a Bayesian deep learning framework for end-to-end classification of multi-class CRC histological images and achieved the highest accuracy of $92.44\%$ following a 10-fold cross-validation scheme^36^, while^37^ proposed an explainable classifier mechanism for ease of human interpretability whose performance was enhanced using a fine-tuned CNN model which obtained an accuracy of $92.74\%$ on the multi-class classification problem. Researchers have also leveraged the concept of transfer learning^38^—the process of fine-tuning a deep learning model pre-trained on a large dataset to suit the given task at hand—to cope with the limited amount of data available in biomedical image analysis tasks. The authors in^39^ explored several deep CNN models pre-trained on the ImageNet dataset^12^ for feature extraction from the histological images. That was followed by multi-class classification using a pool of traditional classifiers like the random forest, Multi-layer Perceptron, K-nearest neighbours (KNN) and Bayesian classifier. Out of the 108 possible combinations of feature extractors and classifiers, they achieved the highest classification accuracy of $92.08\%$ and an F1-score of $92.12\%$, using a pre-trained DenseNet-169 model as the feature extractor and an SVM as the classifier. In^40^, the authors proposed a semi-supervised algorithm wherein they constructed a hypergraph constructed with the features extracted from the CRC tissue images using a pre-trained VGG-19 network. The features were passed through a feed-forward neural network for multi-class tissue classification. They achieved an accuracy of $95.46\%$ along with an average true positive rate of $94.42\%$. Note that all of the aforementioned studies have used the multi-class CRC tissue dataset by^16^ for training and evaluation.

Ensemble learning^14^ is a strategy that takes into account information obtained from multiple models and combines them at inference time to compute the final predictions. This strategy poses the advantage of capturing potentially diverse information from the base learners as well as improving the generalization capability by reducing the overfitting of the model, especially when the data are scarce. As such, an ensemble of models has been found to perform better than a single neural network model^10,11^ and hence, has been of significant interest among the research community^41,42^. The most common ensemble strategies in the literature apply some fusion schemes on the decision scores obtained by the individual models to produce the final predictions. Dif et al.^43^ proposed a dynamic ensemble learning approach in which the most optimal models were selected from a pool of transfer learning-based CNN models using PSO algorithm^15^, which were then combined using voting and averaging ensemble techniques. The authors in^44^ proposed a two-phase ensemble deep neural network (DNN) framework for end-to-end CRC histology classification and achieved an accuracy of $92.83\%$, while the work done in^45^ proposed two ensemble approaches on the decision scores obtained by four pre-trained deep CNN models of varying architectures, and obtained a mean classification accuracy of $96.16\%$ on the CRC histology dataset^16^.

Although the mentioned ensemble learning approaches have been effective, a major drawback it faces is that training multiple DNN models incurs a large computational cost, which may be infeasible on several occasions such as in a resource-constrained environment. A seminal work that sought to tackle this limitation was proposed by^18^, which introduced the concept of snapshot ensemble–training multiple snapshots of the same DNN during a single run. The authors followed the cyclic learning rate scheduling proposed by^46^ that allowed the DNN model to converge to and escape from multiple local minima in fewer training epochs, ensuring diversity among the snapshot models being trained. Hence, ensemble learning can be leveraged without incurring any additional computational costs of training multiple models; all of the base learners can be generated at the expense of training a single neural network only. Snapshot ensemble techniques have been used by researchers in various sub-domains of biomedical image classification^47–50^.

PSO^15^ is a popular swarm-intelligence based meta-heuristic algorithm that has been leveraged on a wide range of problems pertaining to the optimization paradigm, including continuous optimization^51^, task scheduling^52^, data clustering^53^, image thresholding^54^ and segmentation^55^. Binary variants of PSO^56,57^ have also been introduced and used for FS tasks^58^. The main difference between the binary and continuous variants lies in the use of a transfer function^57^, which maps the continuous search space of PSO to a binary one based on a threshold condition. The primary merits of this meta-heuristic lie in its simple concept, less number of parameters and computational efficiency over other meta-heuristics. These facts have been the key reasons for its successful application over various domains as mentioned.

All of the aforementioned snapshot ensemble methods generate decision scores from the snapshot models and fuse them for the final prediction. In this research, we adopt a different approach, where deep features are extracted from the model snapshots and fused, after which the obtained feature vector is passed through a meta-heuristic, called PSO, for dimensionality reduction. Based on these optimally selected feature subsets, the final classification is performed using the KNN classifier. Since the deep features are extracted from the same CNN model at different learning environments through the training process and fused, there may be redundant features in the feature space that can be eliminated for efficient storage of data. For this, the selection of the optimal feature subset is an important step in the proposed framework. The obtained results on a publicly available CRC dataset by^16^ support the viability of the proposed method.

## Proposed method

In this section, we aptly describe the steps of the proposed framework for CRC histological image analysis. Specifically, the sequential stages of the proposed method are:

1. Transfer learning-based snapshot model training: a pre-trained MobileNet-V2 model is fit on the CRC histological data for fine-tuning the model.
2. Feature extraction and fusion from model snapshots: while training the MobileNet-V2 model at certain epochs, the features are extracted from the penultimate fully connected layer of the model. These features are then concatenated to yield the ensembled feature vectors.
3. FS using PSO algorithm: upon the concatenated features, PSO algorithm is used for dimensionality reduction of the feature set and removal of the redundant features for the classification.

### Transfer learning-based snapshot model training

**Figure 2:**
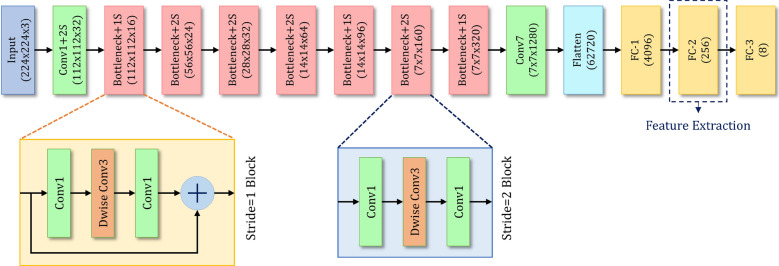
Architecture of the customized MobileNet-V2 CNN model used in this research.

The motivation behind the transfer learning approach is to fine-tune a DNN pre-trained on a large dataset, like ImageNet^12^, for classification on the dataset of the current problem which consists of a limited amount of data. We use the MobileNet-V2^13^ CNN model pre-trained on the ImageNet^12^ dataset as the backbone of our framework. Proposed by^13^, MobileNet-V2 is a lightweight state-of-the-art CNN model developed as an improvement upon its predecessor^59^. With the introduction of inverted residual blocks instead of the conventional residual blocks^60,61^, and depth-wise separable convolutions, which are the combination of the depth-wise and point-wise convolution, the number of parameters and thereby, the computational cost of MobileNet-V2 is greatly reduced when compared to the other DCNN architectures^59,60,62^.

To capture the information in an effective way from the histological images using the pre-trained MobileNet-V2 model, two fully connected (FC) layers have been added after flattening the final Rectified Liner Unit—$\hbox {ReLU}6$ layer of the base CNN. The flattened layer consists of 62720 units and directly mapping them to the final classification layer may lose important information. For this, we introduce two intermediate FC layers to capture the important information before mapping the same to the classification layer. The first customized FC layer (FC–1) comprises 4096 neurons and the second layer (FC–2) comprises 256 neurons, following which is the final classification layer. Both the layers are associated with LeakyReLU^63^ activation function. Both of these FC layers are trained from scratch and the features are extracted from the FC–2 layer. The final classification layer is an FC layer having 8 units (i.e. number of classes of the given dataset) associated with the softmax activation function which maps the input to the respective class probabilities. The architecture of the customized MobileNet-V2 CNN model used in this study is shown in Fig. 2.

The primary need for ensemble learning is the availability of multiple trained deep learning models. However, training a deep CNN model is a time exhaustive process and also requires high computational resources, often making it infeasible to train several models for ensemble learning. To alleviate it, we leverage the snapshot ensemble technique^18^ by training multiple snapshots of the same deep learning network during a single training run of the model.

A fundamental requisition for effective model ensembling is that the individual models should be diverse enough to capture information that is complementary to each other. If this is ensured, it implies that the ensemble framework has taken into account the various facets of the training data and hence, can be able to classify the tissue images with greater accuracy. Further, the diverse nature of the individual models reduces the overfitting of the framework on the training samples. However, as the ensemble proposed in this study comprises snapshots of the same deep learning model over a single training run, the individual models may tend to be similar and lack in the said diversity. To address this potential limitation, an aggressive learning rate schedule is taken^46^ during the training run that causes large changes to the model weights, which in turn enhances diversity among the model snapshots, making the individual models suitable for a robust ensemble framework.

We have used the cosine annealing learning rate scheduler proposed in^46^. The intuition followed here is to accelerate the lowering of the learning rate which forces the model to converge to a local minimum during a cycle of decay. The periodic nature of the cosine function ensures that the learning rate is re-initialized to its initial value at the beginning of each cycle, implying a drastic increase in the learning rate from its previous epoch, which considerably perturbs the weights of the model and thereby allows the model to escape from the minimum it had converged to earlier. The weights of the converged models obtained at the end of each cycle are essentially the “snapshot” base learners constituting the ensemble framework, which are saved and used in subsequent stages.

1 $$\begin{aligned} \alpha (t) = \frac{\alpha _{0}}{2}\left( \cos \left( \frac{\pi mod(t-1,[E/C])}{[E/C]}\right) +1\right) \end{aligned}$$

**Figure 3:**
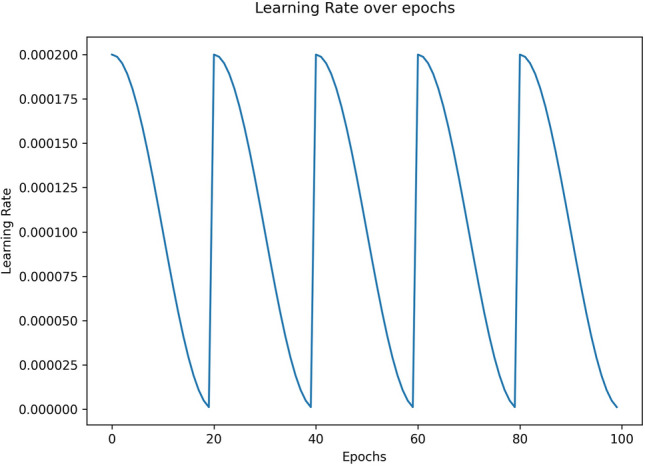
Cyclic variation of learning rate under cosine annealing learning rate schedule over training epochs.

The learning rate $\alpha$ at current epoch (*t*) is given by Eq. (1), where $\alpha _{0}$ is the initial learning rate at the start of training, *E* is the total number of training epochs and *C* is the number of cycles into which the training loop is divided uniformly. In this study, we have trained our model for $E=100$ epochs with $C=5$ cycles, the initial learning rate being set as $\alpha _{0}=2\times 10^{-4}$. Figure 3 shows the variation of learning rate under the aforementioned cyclic learning rate scheduler over training epochs.

### Feature extraction and fusion from model snapshots

After having the generated snapshots from the MobileNet-V2 model, we use them to extract features from both the training and test sets. The features are extracted from the FC–2 layer (described in section “Transfer learning-based snapshot model training”), thereby getting a 256 dimension feature vector for each image from every snapshot model. We stack up the corresponding feature vectors obtained from each snapshot model, thus obtaining a $256\times 5=1280$ dimension fused feature vector for every individual image. We have also taken particular care to ensure that the train-test split is maintained throughout and there is no data leakage.

### Feature selection using PSO

FS is the process of selecting a subset from a set of features such that the most discriminatory features are chosen, thereby enhancing performance and reducing redundancy among the features. Out of a set of *N* features, an exhaustive search for the most optimal subset would incur $2^N$ number of computations, which being exponential is a combinatorially hard problem. This has led to researchers resorting to population-based meta-heuristic search algorithms^27^, which are probabilistic in nature and search over the search space for a near-optimal solution in polynomial time. Due to their stochastic behaviour, require a feasible number of iterations and population size so as to perform an effective search over the domain with a higher probability of finding the global/near optimal solution. In this work, we have adopted the PSO^15^, a popular swarm-based meta-heuristic algorithm to perform FS on the fused feature set (as described in section “Feature extraction and fusion from model snapshots”) so as to eliminate the implicit redundancy that may have crept in due to the use of same CNN backbone over the respective snapshot models.

**Figure 4:**
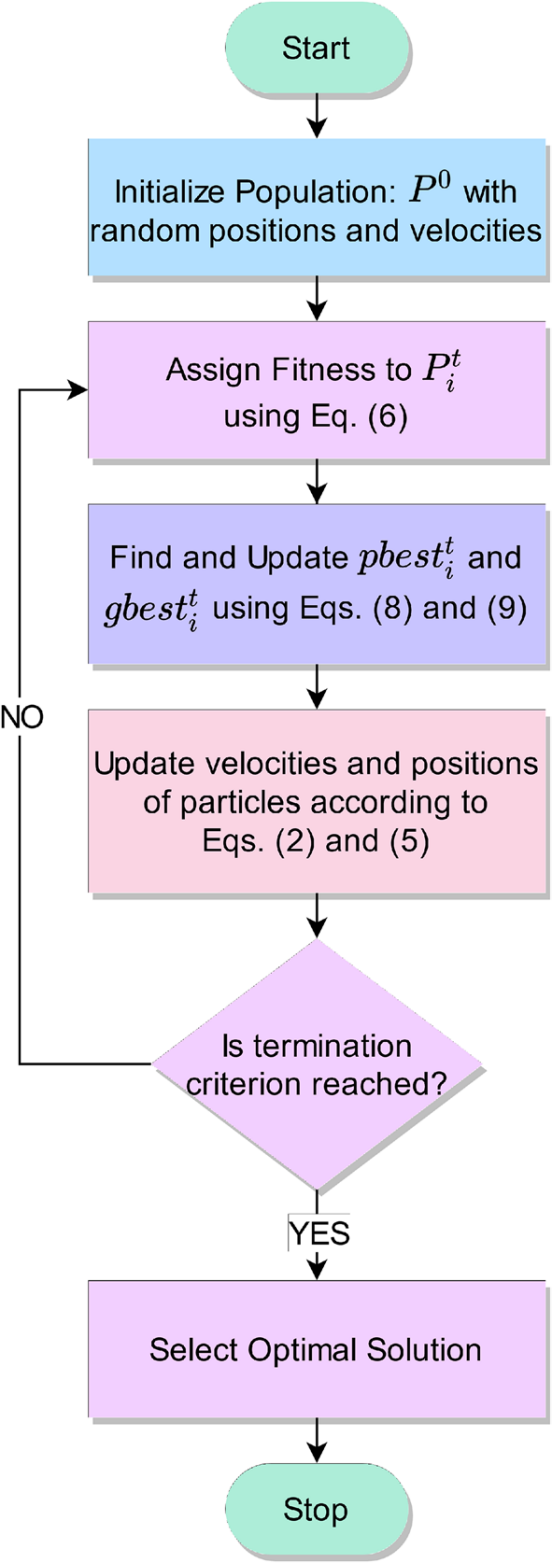
Flowchart of the PSO algorithm.

In a typical PSO setup, the population comprises particles having velocities $v_i$ and positions $x_i$ as their attributes. For current iteration (*t*), if the velocity and position of the particle in the $i\textrm{th}$ dimension are $v_i^{(t)}$ and $x_i^{(t)}$ respectively, then Eq. (2) is used to update the velocities in the $(t+1)^{th}$ iteration.

2 $$\begin{aligned} v_i^{(t+1)} = w^{(t+1)}{v_i^{(t)}} + {C_1}{r_1}(pbest_i^{(t)} - x_i^{(t)}) + {C_2}{r_2}(gbest^{(t)} - x_i^{(t)}) \end{aligned}$$

Here, $pbest_i^{(t)}$ is the best solution that the $i\textrm{th}$ particle has obtained so far, $gbest^{(t)}$ indicates the best solution the swarm has achieved so far. The parameter $w^{(t+1)}$ governs the exploration ability of the population and is updated using Eq. (3), while $r_1$ and $r_2$ are random numbers in the range [0, 1]. The expressions ${C_1}{r_1}\left( pbest_i^{(t)} - x_i^{(t)}\right)$ and ${C_2}{r_2}\left( gbest^{(t)} - x_i^{(t)}\right)$ quantify local intelligence and collaboration of particles, respectively. For this study, the values of the constant parameters have been set experimentally as: $C_1=1, C_2=1$.

3 $$\begin{aligned} w^{(t)} = 1 - \frac{t}{T} \end{aligned}$$

Here, *T* is the maximum number of iterations.

Being originally suited for continuous function optimization, PSO is not directly suitable for a discrete (binary) valued problem such as FS^57^. Thus, we first map the real-valued search space of PSO to [0, 1] using the S-shaped sigmoid transfer function as shown in Eq. (4). After this, a threshold-like operation is employed to yield the desired discrete output, using Eq. (5). By convention, a feature index is set to be “1” if it is selected, “0” otherwise.

4 $$\begin{aligned} \mathscr {S}(x)= & {} \frac{\textrm{1}}{\textrm{1} + e^{-x}} \end{aligned}$$

5 $$\begin{aligned} x_i^{(t+1)}= & {} {\left\{ \begin{array}{ll} 1,\quad rand < \mathscr {S}(v_i^{(t+1)})\\ 0,\quad otherwise \end{array}\right. } \end{aligned}$$

where, *rand* is a random number uniformly distributed between 0 and 1.

Population-based search procedures rely on the use of a fitness function that quantifies the suitability of a particular agent configuration (feature subset in this case). Here, we have formulated our fitness function by aptly combined the classification accuracy (which needs to be maximized) and feature subset cardinality (which needs to be minimized), so as to combine the contrasting objectives in a single fitness function, shown in Eq. (6). Higher the value of the fitness, better is the quality of the feature subset chosen.

6 $$\begin{aligned} \uparrow \mathscr {F} = \omega \times \eta + (1 - \omega ) \times \Delta \end{aligned}$$

where, $\eta$ is the classification accuracy of the feature subset obtained using the embedded KNN classifier^64^, $\Delta$ quantifies the feature reduction given by Eq. 7, and $\omega$ ${\in }$ [0, 1] represents the relative weightage between the classification accuracy and the feature reduction. Following^9^, we have taken $\omega = 0.99$, while the value of ‘k’ for the KNN classifier has been experimentally set to 6.

7 $$\begin{aligned} \Delta = \frac{(|D| - |d|)}{|D|} \end{aligned}$$

where, |*d*| is the number of features selected, and |*D*| is the original feature dimension. In our work, $|D| = 1280$ (as specified in section “Feature extraction and fusion from model snapshots”).

Finally, the local best (*pbest*) and global best (*gbest*) solutions are updated as given by Eqs. (8) and (9) respectively.

8 $$\begin{aligned} pbest_i^{(t+1)}= & {} {\left\{ \begin{array}{ll} x_i^{(t+1)},\quad \mathscr {F}\left( x_i^{(t+1)}\right) > \mathscr {F}\left( pbest_i^{(t)}\right) \\ pbest_i^{(t)},\quad otherwise \end{array}\right. } \end{aligned}$$

9 $$\begin{aligned} gbest^{(t+1)}= & {} {\left\{ \begin{array}{ll} pbest_i^{(t+1)},\quad \mathscr {F}\left( pbest_i^{(t+1)}\right) > \mathscr {F}\left( gbest^{(t)}\right) \\ gbest^{(t)},\quad otherwise \end{array}\right. } \end{aligned}$$

where, $\mathscr {F}(\cdot )$ is the fitness function defined in Eq. (6). Figure 4 shows the flowchart of the PSO algorithm used in this research.

### Statement

All experiments and methods were carried out in accordance with relevant guidelines and regulations.

## Results and discussion

In this section, we provide details of the dataset used for evaluating our proposed method, as well as show and compare the results obtained with existing state-of-the-art approaches in literature to justify the superiority and reliability of the proposed method.

### Dataset description

In this study, we have used the CRC histology dataset by^16^, a collection of textures in histological images of human CRC containing 5000 images of $150 \times 150$ px each ($74\times 74$ $\upmu \hbox {m}$). Each image belongs to exactly one of eight tissue categories as given in Table 1. The dataset is class-balanced with each class comprising 625 images. Following a 5-fold cross-validation scheme for our experiments, we split each tissue category into 500/125 images for train/test respectively.

### Evaluation metrics

Four commonly used metrics, namely Accuracy, Precision, Recall and F1-Score, have been employed for evaluation of the proposed method on the publicly available CRC histology dataset^16^.

For a multi-class system (*N*-class), if we have a confusion matrix *M*, with the rows depicting the predicted class and columns depicting the true class, these evaluations metrics can be formulated as in Eqs. (10)–(13).

10 $$\begin{aligned} Accuracy= & {} \frac{\sum _{i=1}^{N}M_{ii}}{\sum _{i=1}^{N}\sum _{j=1}^{N}M_{ij}} \end{aligned}$$

11 $$\begin{aligned} Precision_{i}= & {} \frac{M_{ii}}{\sum _{j=1}^{N}M_{ji}} \end{aligned}$$

12 $$\begin{aligned} Recall_{i}= & {} \frac{M_t{ii}}{\sum _{j=1}^{N}M_{ij}} \end{aligned}$$

13 $$\begin{aligned} F1-Score_{i}= & {} \frac{2}{\frac{1}{Precision_{i}}+\frac{1}{Recall_{i}}} \end{aligned}$$

### Implementation

**Table 2:** Results obtained by the proposed method on the 5-folds of cross validation on the CRC histology dataset.

Fold	Accuracy (%)	Precision (%)	Recall (%)	F1-Score (%)
1	97.60	97.64	97.60	97.61
2	97.60	97.64	97.60	97.61
3	97.50	97.54	97.50	97.51
4	97.60	97.64	97.60	97.61
5	97.70	97.74	97.70	97.71
Avg.± Std. Dev.	97.60 ± 0.07	97.64 ± 0.07	97.60 ± 0.07	97.61 ± 0.07

**Figure 5:**
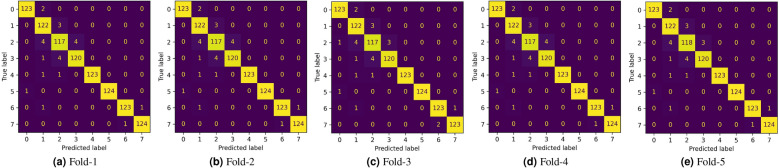
Confusion matrices obtained by the proposed method on the 5-folds of cross validation on the CRC histology dataset.

**Figure 6:**
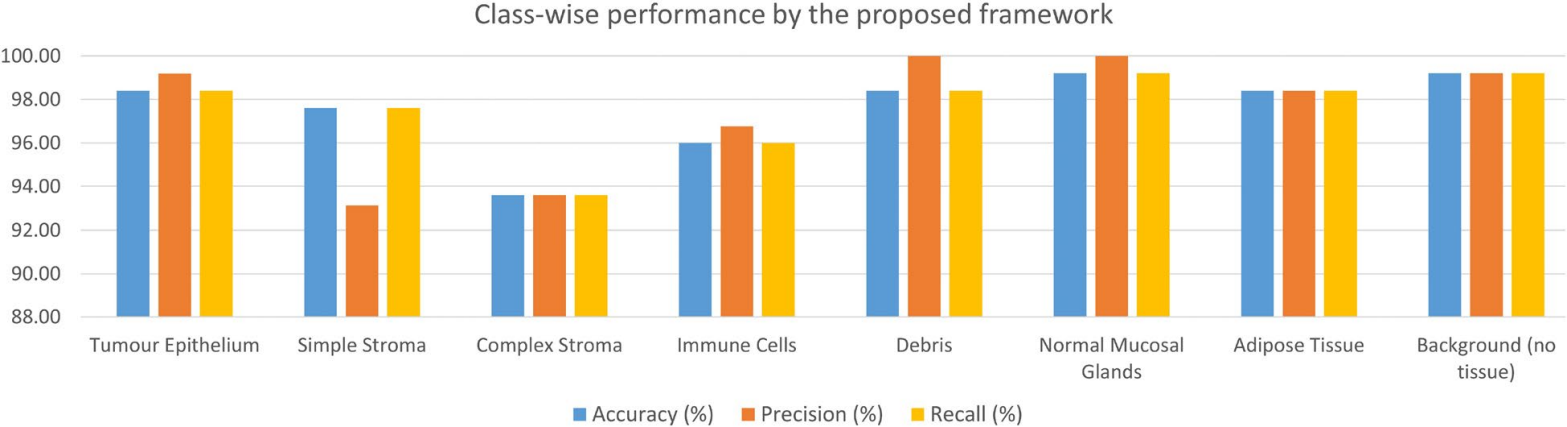
Class-wise results obtained by the proposed method on the CRC histology dataset. The average performance over the 5 folds of cross-validation are reported.

**Table 3:** Results obtained from the transfer learning phase by training various CNN models on the said dataset for 100 epochs; the average values over 5-folds of cross validation are reported.

CNN Model	Accuracy (%)	Precision (%)	Recall (%)	F1-score (%)
AlexNet^65^	96.30	96.40	96.30	96.32
Wide-ResNet-50-2^61^	95.40	95.40	95.35	95.37
VGG-19^66^	95.25	95.25	95.30	95.27
DenseNet-201^67^	95.75	95.80	95.70	95.72
MobileNet-V2^13^	96.60	96.65	96.60	96.61

**Table 4:** Accuracy (%) obtained by each snapshot model during training the base CNN on each fold of 5-fold cross validation.

Snapshot Model	Fold 1 (%)	Fold 2 (%)	Fold 3 (%)	Fold 4 (%)	Fold 5 (%)
Snapshot 1	94.80	94.60	94.50	95.00	95.00
Snapshot 2	96.40	96.00	95.30	94.90	95.30
Snapshot 3	95.60	95.80	95.60	96.00	96.00
Snapshot 4	96.60	95.90	95.90	96.60	96.50
Snapshot 5	95.80	96.00	96.00	96.00	96.60

**Figure 7:**
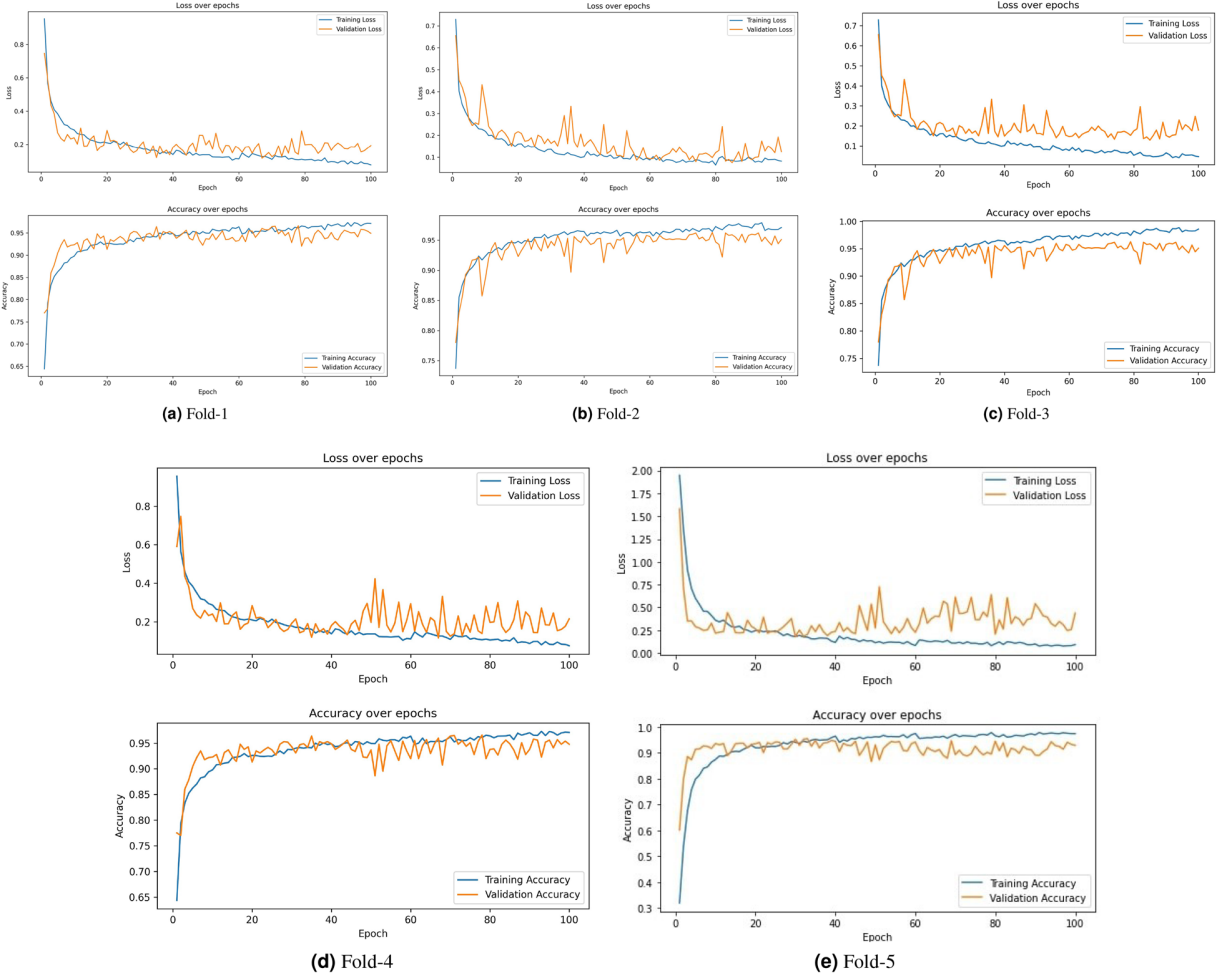
Learning curves obtained by the base CNN model of the proposed method on the 5-folds of cross validation on the CRC histology dataset.

**Table 5:** Comparison of results obtained on performing FS on the ensembled feature set; the average values over 5-folds of cross validation are reported.

FS Algorithm	Accuracy (%)	Precision (%)	Recall (%)	F1-Score (%)	FS (out of 1280)
GWO^68^	97.40	97.44	97.40	97.41	727
SCA^69^	97.40	97.44	97.40	97.41	502
GSA^70^	97.50	97.54	97.50	97.51	665
CSA^71^	97.30	97.33	97.30	97.31	457
WOA^72^	97.40	97.44	97.40	97.41	508
PSO^57^	97.60	97.64	97.60	97.61	592

The proposed framework has been implemented in PyTorch^73^ on a 8GB Nvidia GeForce 2080 GPU. The base snapshot CNN was trained for 100 epochs with 5 cycles comprising 20 epochs each (as discussed in section “Transfer learning-based snapshot model training”) with an initial learning rate of $2\times 10^{-4}$ using the Stochastic Gradient Descent^74^ optimizer. All histological images were resized to $224\times 224$ using bilinear interpolation before being fed into the CNN.

In this study, we have employed a 5-fold cross-validation scheme for evaluating the proposed pipeline on the CRC histology dataset^16^. The results obtained by the proposed method on each fold of the cross-validation along with the average and standard deviation values over the five folds are tabulated in Table 2. Further, the performance of the proposed method on each class of the dataset (described in Table 1) is shown in

- Figure 5 which comprises the confusion matrices obtained on each fold of cross-validation, and
- Figure 6 which gives a graphical representation of the class-wise metric scores averaged over the five folds of cross-validation.

For the transfer learning phase, different state-of-the-art pre-trained CNN models are customized and fine-tuned on the given dataset, the results of which are tabulated in Table 3. We can see that MobileNet-V2^13^ shows the best performance among all the CNN models, while AlexNet^65^ achieves comparable performance to the former. Owing to its superior performance, we justify the usage of MobileNet-V2 as the base CNN model to be used for deep snapshot ensembling.

Table 4 tabulates the validation accuracies obtained by individual snapshot models during deep snapshot training on each fold of the 5-fold cross-validation scheme. The learning curves obtained during the CNN model training process over 100 epochs for each fold are shown in Fig. 7. Most of the learning curves show a gradual decrease in the losses (and a similar rate of increase in accuracy), ensuring that the models have converged effectively and are not overfitted, except that in Fold-3, the somewhat divergent behaviour of the training and validation loss curves do depict a comparatively weaker learning behaviour.

PSO algorithm^57^ has been adopted in this study to select the most optimal features from the fused feature set obtained from the respective snapshot models. To justify its use quantitatively, it has been compared with the following state-of-the-art metaheuristic optimization algorithms in literature:

1. Grey Wolf Optimizer (GWO)^68^
2. Sine Cosine Algorithm (SCA)^69^
3. Gravitational Search Algorithm (GSA)^70^
4. Cuckoo Search Algorithm (CSA)^71^
5. Whale Optimization Algorithm (WOA)^72^

For every fold, each of the algorithms is run separately for 10 times on the fused feature set with and the average values of the evaluation metrics are considered. For each run, the maximum number of iterations for the FS algorithm is set to 50. This is done to ensure robustness in the performance of the algorithms, as they are stochastic in nature. The results of the aforementioned comparative study are tabulated in Table 5. It can be observed that PSO shows the best performance in terms of all the evaluation parameters and is also found to show very competitive performance in terms of the number of selected features. GSA ranks second in terms of the metric values, whereas CSA is found to select the minimal number of features. The results justify the effectiveness of using PSO for FS in the proposed study.

### Comparison with state-of-the-art methods

Table 6 compares the results obtained by the proposed framework against existing state-of-the-art methods for CRC tissue classification. It can be observed that the proposed method outperforms all of the existing works in literature by a significant margin in terms of all the four evaluation metrics used in this study. Further, several of the methods in the literature have mentioned accuracy as their sole evaluation metric, which does not provide information about false positives (or true negatives) and thereby is not a sufficient parameter to evaluate a multi-class classification framework. On the other hand, our results justify that the proposed study is a highly effective and superior approach in CRC detection from the histological analysis.

Most of the existing methods on the CRC dataset^16^ use a single deep learning model for the classification of the histological slide images. Among the compared methods shown in Table 6, only^45^ explored the ensemble learning approach by leveraging a simple probability averaging ensemble of decision scores, and their performance ranks closest to that obtained by the proposed method (96.16% accuracy). However, this work used multiple CNN classifiers to form the ensemble making them computationally expensive. In contrast, our proposed method requires the training of one CNN model for the ensemble, and still outperforms^45^ by a fair margin.

**Table 6:** Comparison of the proposed framework with state-of-the-art methods on CRC histology dataset^16^ used in this study.

Method	Accuracy (%)	Precision (%)	Recall (%)	F1-Score (%)
Kather et al.^16^	87.40	–	–	–
Ciompi et al.^35^	79.66	–	–	–
Wang et al.^34^	92.60	–	92.80	–
Rkaczkowski et al.^36^	92.44	–	–	–
Dif et al.^43^	94.52	–	–	–
Sabol et al.^37^	92.74	92.50	92.76	92.64
Ohata et al.^39^	92.08	–	–	92.12
Paladini et al.^45^	96.16	–	–	–
Bakht et al.^40^	95.46	–	–	94.00
Ghosh et al.^44^	92.83	92.83	93.11	92.97
Marik et al.^75^	95.22	95.34	95.22	95.26
Proposed method	97.60	97.64	97.60	97.61

### Ablation study

To investigate the use of snapshot feature fusion over cyclic learning rate scheduling episodes, we compare its performance against baseline setups obtained on conducting an ablation study on the framework. For each of the baselines, a 5-fold cross-validation scheme is adopted during experimentation.

- **B1**: Features are extracted using the base CNN model without any fusion, the rest of the experimental setup remains unaltered. For this setup, the dimension of extracted feature vector has been set to 512.
- **B2**: Features are extracted using the top-2 performing snapshot models and fused, keeping the rest of the experimental setup unchanged (i.e. output feature dimension is 512).
- **B3**: Features are extracted using the top-3 performing snapshot models and fused, without any changes in the rest of the experimental setup.
- **B4**: Features are extracted using the top-4 performing snapshot models and fused without altering the rest of the experimental setup.

**Figure 8:**
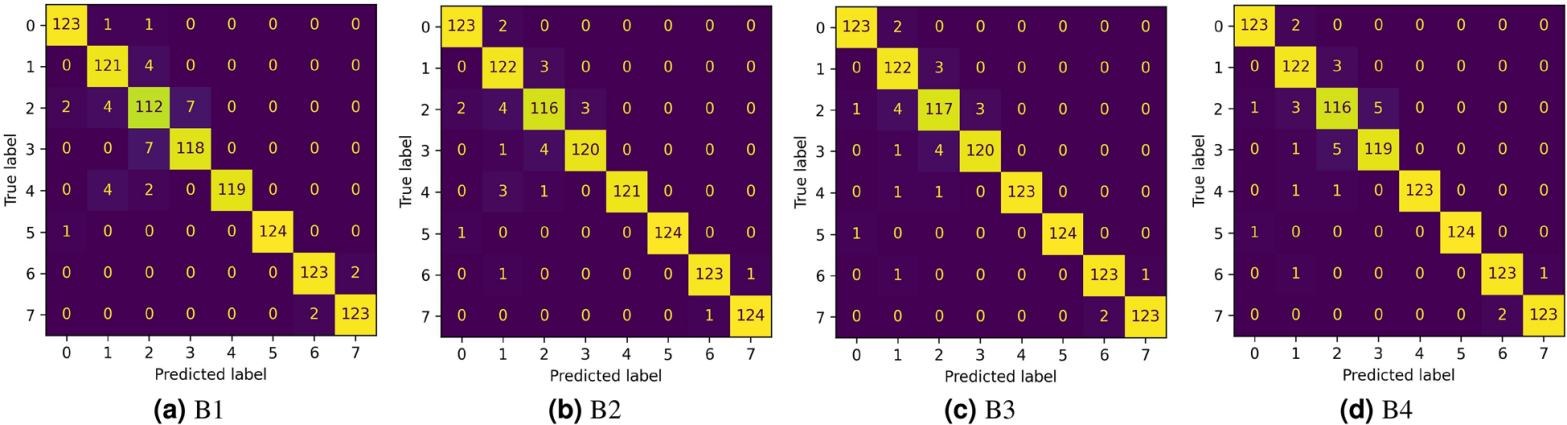
Confusion matrices obtained from each baseline setup as described in section “Ablation study”.

Table 7 tabulates the results of the aforementioned comparative study. It can be observed that the proposed approach outperforms all of the chosen baseline setups in terms of all the evaluation metrics. Baselines **B2** and **B4** show very similar performance in terms of accuracy and F1-Score, with a marginal difference in precision values. The setup **B3** performs best among the chosen baselines, although it falls short as compared to the proposed method. Further, the fact that **B1** shows the weakest performance, highlights the superiority of ensemble learning over a single network. The confusion matrices depicting class-wise performances for each baseline setup are shown in Fig. 8.

**Table 7:** Results of the Ablation study obtained from each setup as described in section “Ablation study”; the average values over 5-folds of cross-validation are reported.

Baseline	Feature Dimension	Accuracy (%)	Precision (%)	Recall (%)	F1-Score (%)
B1	512	96.30	96.35	96.30	96.32
B2	512	97.30	97.36	97.30	97.31
B3	768	97.50	97.54	97.50	97.51
B4	1024	97.30	97.33	97.30	97.31
Proposed method	1280	97.60	97.64	97.60	97.61

### Analysis of the proposed model

In this section, we thoroughly analyse the performance of the proposed model both quantitatively and qualitatively so as to prove its robustness and justify the significance of the results obtained.

#### Statistical test

To statistically analyze the significance of the proposed method, the McNemar’s statistical test^76^ is performed between the PSO algorithm used in the proposed method and other popular metaheuristics used in the comparisons. McNemar’s test is a non-parametric test^77,78^, which assumes the null hypothesis that two models are statistically similar. To reject this null hypothesis, the *p*-value obtained must be lower than $5\%$. The results of McNemar’s test are shown in Table 8. It can be noted that for every scenario, $p-value<0.05$, and thus the null hypothesis is rejected, justifying that the proposed model is statistically dissimilar to other methods.

**Table 8:** Results (in terms of *p*-values) attained by applying McNemar’s statistical test between PSO algorithm used in the proposed model versus other popular metaheuristic algorithms (GWO, Grey Wolf Optimizer; SCA, Sine-Cosine Algorithm; GSA, Gravitational Search Algorithm; CSA, Cuckoo Search Algorithm; WOA, Whale Optimization Algorithm).

McNemar’s test	*p*-value
PSO vs. GWO	3.53E−02
PSO vs. SCA	2.81E−02
PSO vs. GSA	2.95E−02
PSO vs. CSA	1.17E−02
PSO vs. WOA	3.48E−02

#### t-SNE visualization

**Figure 9:**
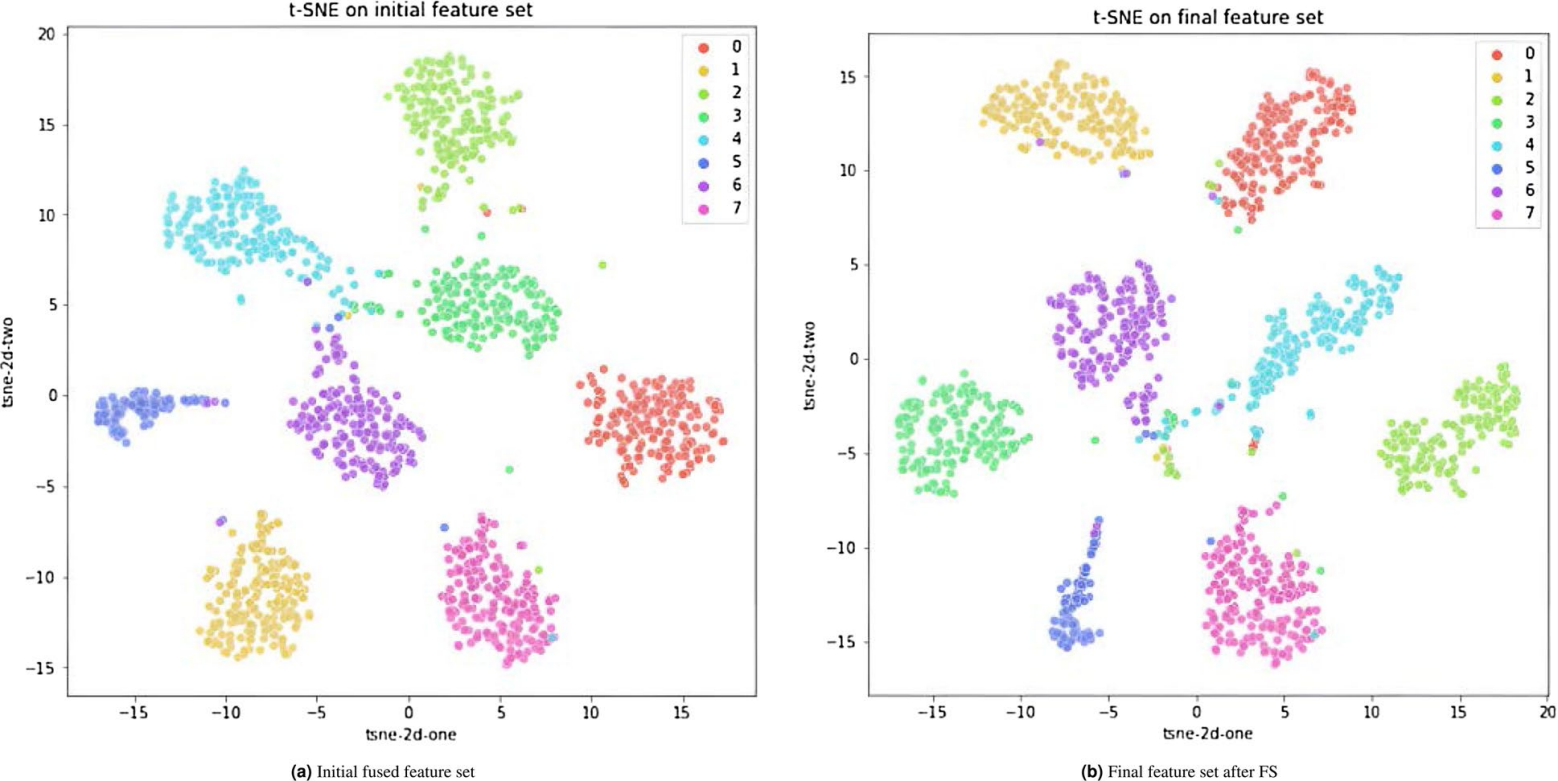
$t-$SNE visualization of (**a**) the snapshot fused features obtained from base model training and (**b**) the final feature subset obtained by the full framework.

The $t-$Distributed Stochastic Neighbourhood Embedding (or simply $t-$SNE) is a dimensionality reduction algorithm^79^ that converts high-dimensional dataset into a matrix of pairwise similarities, which can be subsequently visualized in 2D (or 3D) using suitable tools. In this paper, we have employed $t-$SNE to the snapshot fused feature set and that obtained after FS, and visualized the low-dimensional resultant in a 2D scatter-plot, as shown in Fig. 9. We can observe in both figures that the features are mostly well-clustered in their own classes, as well as separated apart from other classes. Furthermore, the class-wise separation in the final feature subset is more prominent compared to the initial feature set, thus highlighting the usefulness of FS and justifying its role in boosting performance. Thus, we can conclude that the snapshot fusion technique enables the formation of a highly discriminative embedding space, which justifies the high performance of our model.

#### Grad-CAM analysis

**Figure 10:**
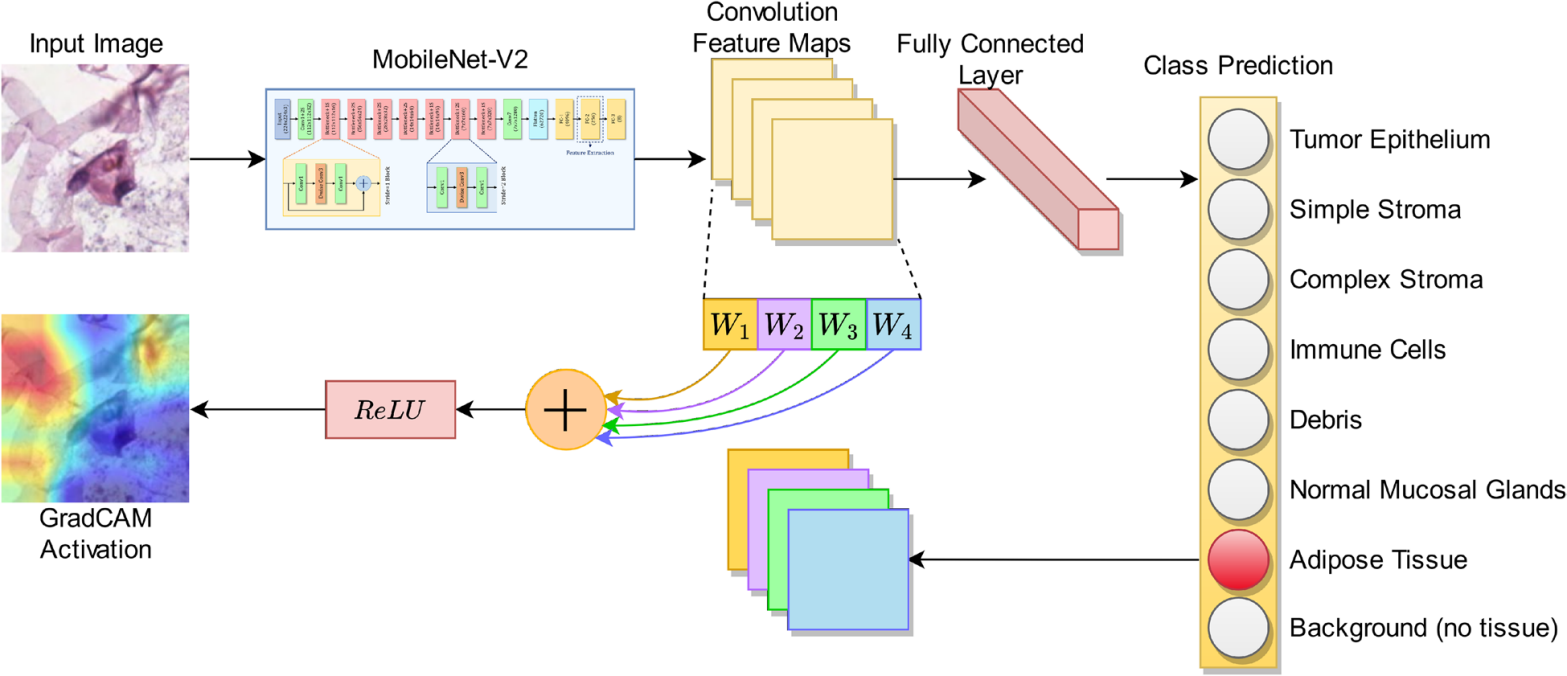
Explanation of the Grad-CAM activation process.

**Figure 11:**
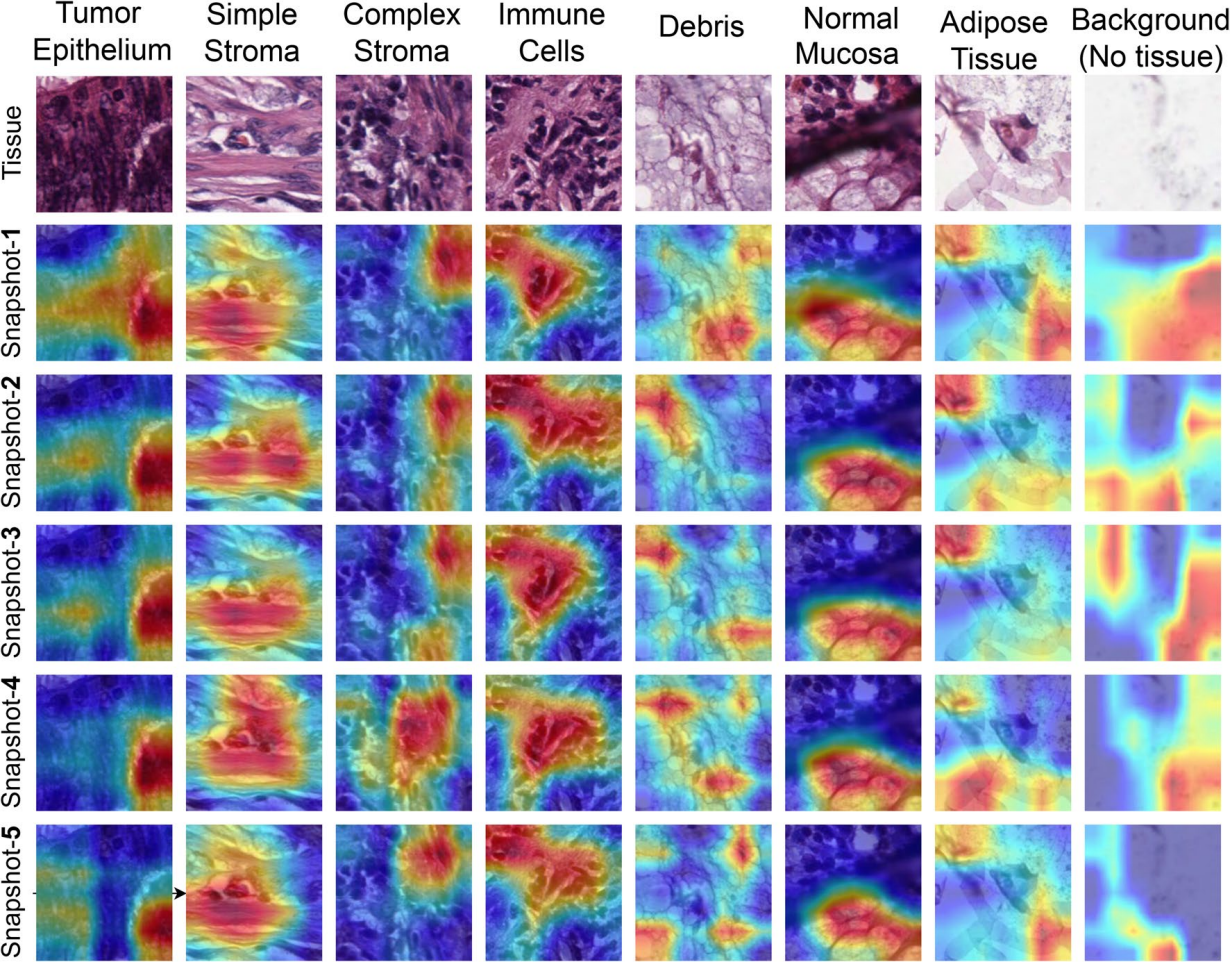
Grad-CAM activations obtained on some samples taken from the CRC dataset^16^ at different snapshots are shown. As can be seen, there is both redundancy as well as diversity among regions activated by the network, which qualitatively explains the benefit of snapshot ensembling and as well as need for FS.

Gradient-weighted Class Activation Mapping (Grad-CAM) is a class-discriminative localization process introduced by^17^ that produces a visual representation of the regions of a given image identified by the CNN model to be most distinguishing and thereby, highly pertinent to its class prediction. The process followed is that the input image is first passed through the CNN model for label prediction, after which the weighted average of the activation maps is taken from the last convolutional layer of the network to form the activation heat map, which is then superimposed on the original image to highlight the distinct regions of particular interest shown by the model for the classification task. An explanatory diagram for this process is illustrated in Fig. 10.

We have used Grad-CAM analysis on each of the snapshot models obtained during the training to investigate the regions focused on by each snapshot model on a given CRC histological image, which would give insights into the information captured by the individual snapshots. Figure 11 shows the Grad-CAM visualisations using each of the snapshot models on correctly classified sample tissue images from the testing samples of the CRC histology dataset^16^ used in this study. It is observed that different snapshots have generally been shown to have activated diverse regions of the original image of most classes, implying that complementary information has been extracted by the different snapshots leading to the success of the fusion framework. Further, for some of the classes (i.e. Tumour Epithelium and Simple Stroma), all of the snapshot models seem to have captured almost the same regions of the original image, showing little diversity. This implies that a considerable percentage of the information is redundant after fusing the features, which in turn justifies the inclusion of FS in the proposed framework.

### Additional testing

To further validate the applicability and robustness of the proposed method, we additionally test it on the publicly available LC25000 dataset^80^, which consists of histopathology images of the human lung and colon for detection of cell carcinoma and adenocarcinoma. The lung dataset has 3 class while the colon dataset has 2 classes, with each class comprising 5000 images. Since our work focuses on colorectal histological analysis, we evaluate our method on the 2-class colon dataset only, the classes, the classes being “normal” and “adenocarcinoma”.

Keeping all elements of the experimental protocol identical (i.e., the same hyperparameters, five-fold cross-validation with 8000 and 2000 train and test images, respectively etc.), we test our proposed framework on the colon dataset^80^. The result (accuracy in %) obtained along with a comparison to state-of-the-art methods found in literature has been shown in Table 9. As evident from the empirical values, our model achieves a high accuracy of $99.99\%$, surpassing all prior state-of-the-arts^81–84^ on the said dataset by a significant margin, thus proving its applicability across CRC datasets.

**Table 9:** Comparison of accuracy scores attained by the proposed model against prior state-of-the-art methods on the colon dataset taken from the LC25000 dataset^80^.

Method	Description	Accuracy (%)
Liang et al.^81^	Multi-scale feature fusion with shearlet transform	96.00
Mangal et al.^82^	Shallow CNN	96.00
Qasim et al.^83^	Lightweight and fast CNN	99.60
Yildirim et al.^84^	A CNN variant	99.75
Proposed	FS-aided snapshot ensemble	99.99 ± 0.002

For the sake of convenience, we have included a one-liner description of each of the comparative methods.

**Figure 12:**
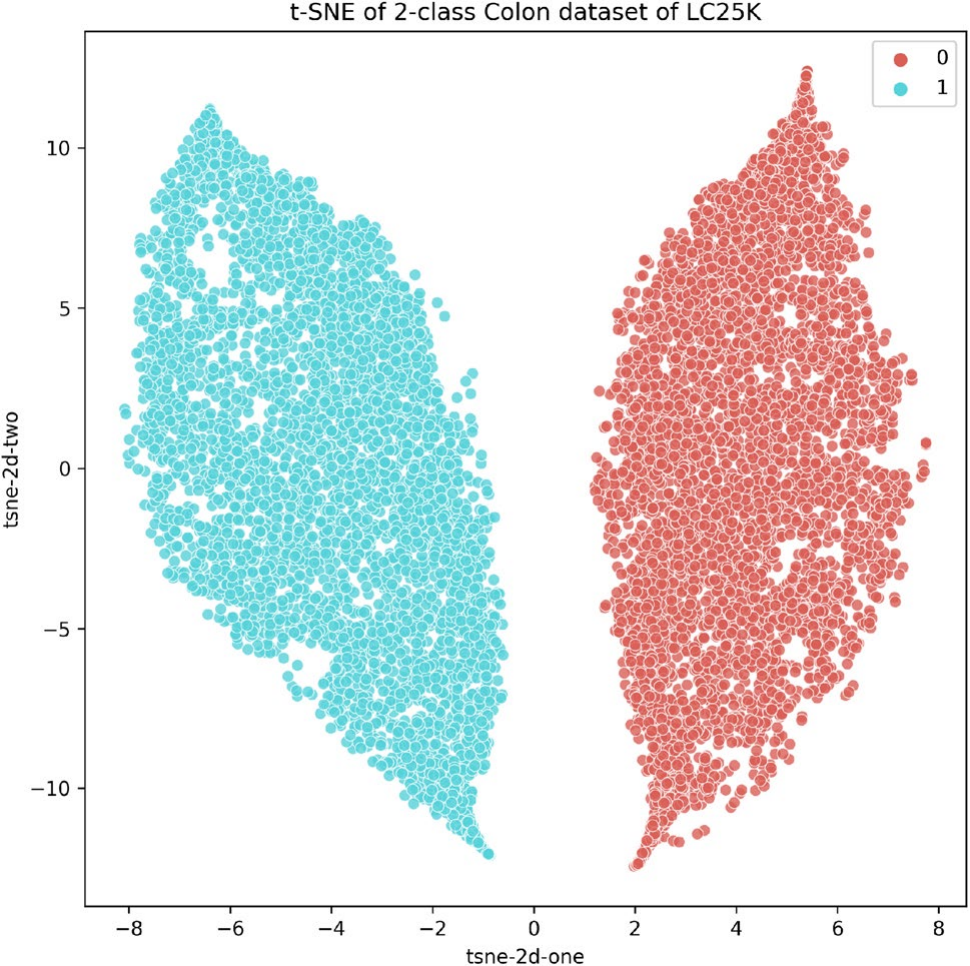
$t-$SNE visualization of features obtained by the proposed framework on the LC25000 Colon dataset^80^.

Furthermore, we have applied $t-$SNE^79^ on the features obtained by the proposed model on the colon dataset to visualize the embedding space in a 2D plot, depicted in Fig. 12. As expected, based on the near-perfect results obtained, the two classes are highly segregated apart without any overlap of samples. This qualitatively conforms to the empirical results reported in Table 9 and further justifies the robustness of our approach on generalizing to other dataset.

## Conclusion

CRC accounts for more than 800 K deaths annually and is the third most common type of cancer diagnosed. The detection of CRC requires expert pathologists to classify each cell from histological slides of the colon. Such a tedious and expensive process hinders the population-wide screening and thus often the disease is diagnosed at a much later stage. Hence, Computer-Aided Detection frameworks are being developed for the automated and early diagnosis of the disease. In this research, we use a deep learning-based approach to detect CRC from histological slide images. Specifically, we apply an ensemble model for the disease prediction, where instead of fusing different base learners, we use one single CNN model and take model snapshots at different epochs and form an ensemble. This is computationally much cheaper than fusing two or more base learners since the training process is undergone only once. However, in literature, snapshot ensemble has been performed in other domains by fusing the decision scores obtained at the different checkpoints. In this research, we adopt a different approach, wherein we extract deep features from the penultimate FC layers of the MobileNet-V2 CNN model to form the ensemble. The extracted features from the different model snapshots are fused and binary PSO is employed to reduce the dimensionality of the feature space, eliminating the redundant features and for the final classification. We have obtained a feature reduction of $53.75\%$ (592 features selected out of 1280), which implies that redundant information has been eliminated sufficiently from the fused feature space. The proposed framework applied on a publicly available CRC dataset^16^ outperforms state-of-the-art methods on the same, justifying the reliability of the model. Grad-CAM analysis on the different model snapshots provides for visual explainability of the pipeline and also shows that complementary information is supplied by the CNN model at those snapshots which are efficiently fused by the proposed learning process.

In this work, we have used the cosine annealing learning rate scheduler. In future, we may try other methods for the same. Also, for the FS stage, we may try improving the PSO algorithm by, for example, by adding local search methods or initializing with a guided population, etc. The developed framework is domain-independent, and thus may be applied to other image classification problems as well. In future, we may also try to perform segmentation on the histology images before classification, since an RoI localization may help the medical experts to identify abnormalities in the cells more easily. As seen from the class-wise results obtained by the proposed framework in Fig. 6, relatively poor performance is obtained in some classes like the “Complex Stroma”. From the confusion matrices, it can be seen that some instances from this class are classified into “Simple Stroma” or “Immune Cells”. We may try to analyse and alleviate this problem in the future.

## Data Availability

No datasets are generated during the current study. The datasets analyzed during this work are made publicly available in this published article.

## Code availability

Our source codes are publicly accessible at: https://github.com/soumitri2001/SnapEnsemFS.
